# Histone deacetylase inhibition synergistically enhances pemetrexed cytotoxicity through induction of apoptosis and autophagy in non-small cell lung cancer

**DOI:** 10.1186/1476-4598-13-230

**Published:** 2014-10-09

**Authors:** Donatella Del Bufalo, Marianna Desideri, Teresa De Luca, Marta Di Martile, Chiara Gabellini, Valentina Monica, Simone Busso, Adriana Eramo, Ruggero De Maria, Michele Milella, Daniela Trisciuoglio

**Affiliations:** Experimental Chemotherapy Laboratory, Regina Elena National Cancer Institute, Rome, Italy; Department of Oncology, University of Torino, AOU San Luigi Gonzaga, Turin, Italy; Department of Hematology, Oncology and Molecular Medicine, Istituto Superiore di Sanità, Rome, Italy; Scientific Direction, Regina Elena National Cancer Institute, Rome, Italy; Division of Medical Oncology, Regina Elena National Cancer Institute, Rome, Italy

**Keywords:** HDAC inhibitors, ITF2357, Givinostat, Pemetrexed, Apoptosis, Autophagy, Synergism, NSCLC

## Abstract

**Background:**

Non-small cell lung cancer (NSCLC) is the leading cause of cancer-related death worldwide. Pemetrexed, a multi-target folate antagonist, has demonstrated efficacy in NSCLC histological subtypes characterized by low thymidylate synthase (TS) expression. Among many other potential targets, histone deacetylase inhibitors (HDACi) modulate TS expression, potentially sensitizing to the cytotoxic action of anti-cancer drugs that target the folate pathway, such as pemetrexed. Since high levels of TS have been linked to clinical resistance to pemetrexed in NSCLC, herein we investigated the molecular and functional effects of combined pemetrexed and ITF2357, a pan-HDACi currently in clinical trials as an anti-cancer agent.

**Results:**

In NSCLC cell lines, HDAC inhibition by ITF2357 induced histone and tubulin acetylation and downregulated TS expression at the mRNA and protein level. In combination experiments *in vitro* ITF2357 and pemetrexed demonstrated sequence-dependent synergistic growth-inhibitory effects, with the sequence pemetrexed followed by ITF2357 inducing a strikingly synergistic reduction in cell viability and induction of both apoptosis and autophagy in all cell line models tested, encompassing both adenocarcinoma and squamous cell carcinoma. Conversely, simultaneous administration of both drugs achieved frankly antagonistic effects, while the sequence of ITF2357 followed by pemetrexed had additive to slightly synergistic growth-inhibitory effects only in certain cell lines. Similarly, highly synergistic growth inhibition was also observed in patient-derived lung cancer stem cells (LCSC) exposed to pemetrexed followed by ITF2357. In terms of molecular mechanisms of interaction, the synergistic growth-inhibitory effects observed were only partially related to TS modulation by ITF2357, as genetic silencing of TS expression potentiated growth inhibition by either pemetrexed or ITF2357 and, to a lesser extent, by their sequential combination. Genetic and pharmacological approaches provided an interesting link between the autophagic and apoptotic pathways, and showed that sequential pemetrexed/ITF2357 causes a toxic form of autophagy with consequent activation of a caspase-dependent apoptotic program. *In vivo* experiments in NSCLC xenografts confirmed that sequential pemetrexed/ITF2357 is feasible and results in increased inhibition of tumor growth and increased mice survival.

**Conclusions:**

Overall, these data provide a strong rationale for the clinical development of sequential schedules employing pemetrexed followed by HDACi in NSCLC.

**Electronic supplementary material:**

The online version of this article (doi:10.1186/1476-4598-13-230) contains supplementary material, which is available to authorized users.

## Background

Lung cancer is the most common cause of cancer-related death worldwide, with non-small cell lung cancer (NSCLC) accounting for approximately 80% of all lung cancers [1, 2]. Despite progresses in systemic treatment, only a small proportion of patients diagnosed with metastatic disease survive over 5 years. Most patients relapse within 1 year of starting first-line treatment mainly due to intrinsic or acquired resistance to chemotherapy [3]. Pemetrexed (Alimta, Eli Lilly and Company), a multi-target folate antagonist, has recently become the cornerstone of treatment for non-squamous carcinoma of the lung [4]. The selective sensitivity of non-squamous NSCLC to pemetrexed cytotoxicity is thought to be related to the levels of expression of thymidylate synthase (TS), an essential enzyme for the *de novo* synthesis of thymidylate and subsequently DNA synthesis, and one of the main intracellular molecular targets of pemetrexed; indeed, elevated TS expression has been proposed as a biomarker of resistance to pemetrexed-based chemotherapy [5–13].

Recently, it has been proposed that patients with NSCLC might benefit from combined treatment with epigenetic drugs [14, 15]. In this context, histone deacetylase inhibitors (HDACi) represent a promising class of antitumor agents, developed to reverse the silencing of critical regulatory pathways [16, 17]. Indeed, the cellular response to treatment with HDACi shows pleiotropic effects involving cell cycle arrest, induction of apoptosis/autophagy and differentiation, modulation of microtubule function, DNA repair, and angiogenesis [18–20]. Based on their ability to activate the apoptotic and autophagic pathways, HDACi may have interest in combination with conventional chemotherapeutic agents to enhance tumor cell chemosensitivity [14, 18, 21, 22]. HDACi have been demonstrated to regulate the expression of several genes and proteins including TS. Transcriptional regulation of TS may be attributed to Rb-E2F1 pathway modulation by p21^waf1⁄cip1^ up-regulation via its promoter histone acetylation by HDACi [23]. Thus, the effects of drugs that critically rely on TS inhibition to exert their cytotoxic action, such as 5-Fluorouracil (5FU), raltitrexed, and pemetrexed [23, 24] can be potentially increased by HDACi [25, 26].

Both apoptosis, a genetically programmed cell death pathway regulated by the complex interaction between anti- and pro-apoptotic proteins, and autophagy, a complex cellular process with a multifaceted role in cell death, have been implicated in the response to antineoplastic treatments [27–29]. Under conditions of limited stress, such as starvation, autophagy promotes cell survival by degrading and recycling long-lived proteins and cellular components [30, 31]; however, when the cell is exposed to prolonged or excessive conditions of stress, autophagy has been shown to result in cell death by self-digestion [32].

In this study we evaluated the antitumor efficacy and the molecular mechanisms of action of ITF2357, a pan-HDACi [33–36], in combination with pemetrexed, using *in vitro* and *in vivo* models of NSCLC and patient derived lung cancer stem-like cells (LCSC). ITF2357 potentiated pemetrexed cytotoxic activity in a sequence-dependent manner: indeed, the combination of pemetrexed followed by ITF2357 showed a highly synergistic interaction *in vitro* in both NSCLC and LCSC cells. The observed decrease in cell viability was due to activation of both apoptosis and autophagy, which were interconnected in the synergistic loss of cell viability induced by sequential pemetrexed/ITF2357.

## Methods

### Cell cultures, plasmids, and transfection

Human NSCLC established cell lines (H1299, H460, A549, H1650, Calu-1) were cultured in 10% inactivated foetal bovine serum (HyClone, Termoscientific, South Logan, UT) in RPMI medium (Invitrogen, Carlsbad, CA). H1299 short hairpin (sh) Beclin1, shControl, EGFP-LC3B and ptf-LC3 stable clones were generated as previously described [37] and cultured in the presence of geneticin (800 μg/ml, Sigma-Aldrich, St. Louis, MO).

Patient-derived LCSC18, LCSC36, LCSC136 and LCSC143 cell lines were isolated as described [38] and cultured in serum-free medium containing 50 *μ*g/ml insulin (Sigma-Aldrich), 100 *μ*g/ml apo-transferrin (Sigma-Aldrich), 10 *μ*g/ml putrescine (Sigma-Aldrich), 0.03 *μ*M sodium selenite (Sigma-Aldrich), 2 *μ*M progesterone (Sigma-Aldrich), 0.6% glucose (Sigma-Aldrich), 5mM HEPES (Euroclone, Pero, Italy), 0.1% sodium bicarbonate (Euroclone), 0.4% BSA (PAA Healthcare, Milan, Italy), glutamine (Euroclone), and antibiotics (Euroclone), dissolved in DMEM–F12 medium (Invitrogen) and supplemented with 20 *n*g/ml EGF (Peprotech, Princeton, NJ) and 10 *n*g/ml bFGF (Peprotech). Flasks non-treated for tissue culture were used to reduce cell adherence and support growth as undifferentiated tumor spheres.

Pooled small interfering RNA (siRNA) oligonucleotides against TS and ATG7 were purchased from Dharmacon RNA Technologies (siGENOME SMART pool, Lafayette, Colorado). For siRNA transfection, cells were seeded and after 24 h transfected with 100 nM pooled oligonucleotides mixture by using Lipofectamine2000 (Invitrogen) following manufacturer’s protocol. 24 h after transfection, media were removed and cells were treated with Pemetrexed (Alimta, formerly LY231514, Eli Lilly and Company) and ITF2357 (Givinostat, Italfarmaco) alone or in combination. Gene silencing efficacy by siRNA was assessed by Western blot or qRT-PCR analyses.

### Reagents preparation and treatments

Cells were treated with ITF2357 and pemetrexed, either alone or in combination, as follows: (a) ITF2357; (b) pemetrexed; (c) ITF2357 and pemetrexed, simultaneously; (d) ITF2357 followed by pemetrexed; (e) pemetrexed followed by ITF2357. Both detached and adherent cells were collected, and differentially processed according to analyses performed.

3-Methyladenine (3MA, 1mM) (Enzo Life Science, Plymouth Meeting, PA, USA), and the pan-caspase inhibitor zVAD-fmk (zVAD, 50μM, Sigma-Aldrich) were dissolved in DMSO. Chloroquine diphosphate (CQ, 25μM, Sigma-Aldrich) was dissolved in water.

### Assessment of cell viability

The inhibitory effect of different drugs was assessed following manufacturer’s protocol on i) NSCLC cell growth by measuring 3-[4,5-dimethylthiazol-2-yl]-2,5-diphenyltetrazolium bromide inner salt (MTT, Sigma-Aldrich) dye absorbance of cells, and ii) LCSC cell growth by quantitation of the ATP present in metabolically active cells using CellTiter-Glo® Luminescent (Promega, Southampton, UK). Data were analyzed by the Chou-Talaly method (CalcuSyn software, Biosoft, Cambridge) to determine the combination index (CI), a well-established index of the interaction between two drugs. CI values of <1, =1, and >1 indicate synergistic, additive, and antagonistic effects, respectively.

### Flow cytometric analysis

Flow cytometric analysis (BD Accuri™ C6, BD biosciences) was performed to evaluate cell cycle distribution by propidium iodide (PI) staining, apoptosis by AnnexinV-FITC/PI staining, and to detect acidic vesicular organelles (AVOs) by acridine orange staining, as previously described [39, 40]. Active Caspase-3 Apoptosis Kit (BD biosciences) was used to detect the heterodimer of 17 and 12 kDa subunits, which is derived from the pro-enzyme.

### Analysis of autophagy

Cells grown on glass coverslips were fixed in 2% formaldehyde for 10 minutes at room temperature. Detection of autophagosomal structures was performed by fluorescence microscopy observing LC3B *puncta* in EGFP-LC3B expressing cells [41]. Autophagic flux was analyzed by fluorescence microscopy monitoring the distribution and alteration of mRFP-GFP-LC3B fluorescent signals [41]. Typically, at least 200 cells were counted, and cells with more than 10 *puncta* were considered autophagy positive. Images were scanned under a × 63 oil immersion objective and, to avoid bleed-through effects, each fluorescent signal was scanned independently by using a Leica DMIRE2 microscope equipped with a Leica DFC 350FX camera, elaborated by a Leica FW4000 deconvolution software (Leica, Solms, Germany) and processed using Adobe PhotoShop software to adjust image brightness and contrast.

### Quantitative real-time polymerase chain reaction (qRT-PCR)

Total RNA was extracted from *in vitro* cultured cells using a Qiagen RNeasy Mini kit (Qiagen) according to the manufacturer’s instructions. Reverse transcription was performed using RevertAid Reverse Transcriptase (Thermo Scientific). qRT-PCR was performed using a Gene-Amp 5700 sequence detection system (Applied Biosystems, Foster City, CA, USA). The mRNA levels were normalized using glyceraldehyde 3-phosphate dehydrogenase (GAPDH), a housekeeping gene that was used as the internal control, because its expression has been demonstrated to remain stable during the protocol. The following primers were used: TS Forward: GCGCTACAGCCTGAGAGATG, TS Reverse: TGCCCCAAAATGCCTCCACT, GAPDH Forward: TCCCTGAGCTGAACGGGAAG, GAPDH Reverse: GGAGGAGTGGGTGTCGCTGT.

qRT-PCR was also performed by using tumor thick sections (10 mm) for RNA extraction. The sections were serial to other 4 mm-thick sections, from the same formalin-fixed paraffin embedded tumor block, used for H&E staining, to select appropriate neoplastic areas and for TS immunohistochemistry. The 10 μm-thick sections were dried overnight at 56°C, de-paraffinated and stained with Nuclear Fast Red solution (Sigma-Aldrich), then rehydrated through graded alcohols and dissected using a scalpel. RNA isolation and retrotranscription were performed as previously reported [42]. An ABI PRISM 7900HT Sequence Detection System (Life Technologies, Applied Biosystems Division, Carlsbad, CA, USA) in 384-wells plate was used. All qPCR mixtures contained 1μl of cDNA template (approximately 40 ng of retrotranscribed total RNA) diluted in 9 μl of distilled-sterile water, 1200 nM of each primer, 200 nM of internal probe and TaqMan Gene Expression Master Mix (Life Technologies) to a final volume of 20 μl. The sequences of primers and probes used for qPCR analyses were previously published [42].

Cycling conditions were 50°C for 2 minutes, 95°C for 10 minutes followed by 46 cycles at 95°C for 15 seconds and 60°C for 1 minute. Baseline and threshold for cycle threshold calculation were set manually with ABI Prism SDS 2.4 Software. A mixture containing Human Total RNA (Stratagene, La Jolla, CA) was used as control calibrator on each plate. β-actin was used as internal reference gene. The fold change in gene expression levels, expressed in unitless values, was evaluated using the 2^–ΔΔCt^ method [43].

### Western blot analysis

Total protein extracts were fractionated by SDS-PAGE, transferred to a nitrocellulose filter and subjected to immunoblot assay. Immunodetection was performed using antibodies directed to: H3 acetylated histone (Millipore, Billerica, MA), TS (ab58287, Abcam), p62/SQTSM1 (Santa Cruz Biotechnology, Santa Cruz, CA), HSP72/73 (Calbiochem, San Diego, CA), LC3B and acetyl-α-tubulin (K40, Sigma-Aldrich), ATG7 (Millipore), caspases 3 and PARP (Santa Cruz). Anti-mouse or anti-rabbit immunoglobulin G (IgG)-horseradish peroxidase conjugated antibodies (Cell Signaling, Amersham Biosciences, Freiburg, Germany) were used as secondary antibodies at 1:10000 dilution. Antibody binding was visualized by enhanced chemiluminescence method (Amersham Biosciences) according to manufacturer’s specification and recorded on autoradiographic film (Amersham Biosciences). Developed films were acquired using GS-700 Imaging Densitometer (Biorad Laboratories, Hercules, CA) and processed with Adobe PhotoShop software. Densitometric evaluation was performed using Molecular Analyst Software (Biorad) and normalized with relative controls depending on the analysis.

### *In vivo*experiments

To evaluate the effect of different treatments on *in vivo* tumor growth, 5 × 10^6^ cells were injected intramuscularly into 6-8 week-old female immunodeficient athymic mice (10 for each group). Weekly intraperitoneal treatment with pemetrexed (1000 mg/Kg), and oral administration with ITF2357 (100 mg/Kg) every 24 h for 4 days started when tumors were palpable (about 7 days after cell injection), and stopped when the animals were sacrificed. Animals were observed daily and tumor volume (mm^3^) calculated as length × width^2^ × *π*/6. Mice survival was calculated by euthanizing the animals when the tumors reached 2.5 g. The experiments were repeated twice. All procedures involving animals and their care were authorized and certified by the decree n. 67/97A of the Italian Minister of Health and protocol 2560/97 of the Rome Health Service Unit (ASL – RMB).

### Statistics

Experiments were replicated three times, unless otherwise indicated, and the data were expressed as mean ± standard deviation (SD) or mean ± standard error (SEM). Differences between groups were analyzed with a two-sided paired or unpaired *t* test and were considered to be statistically significant for p < 0.05.

## Results

### ITF2357 downregulates TS expression and enhances pemetrexed cytotoxicity in NSCLC models

**Figure 1:**
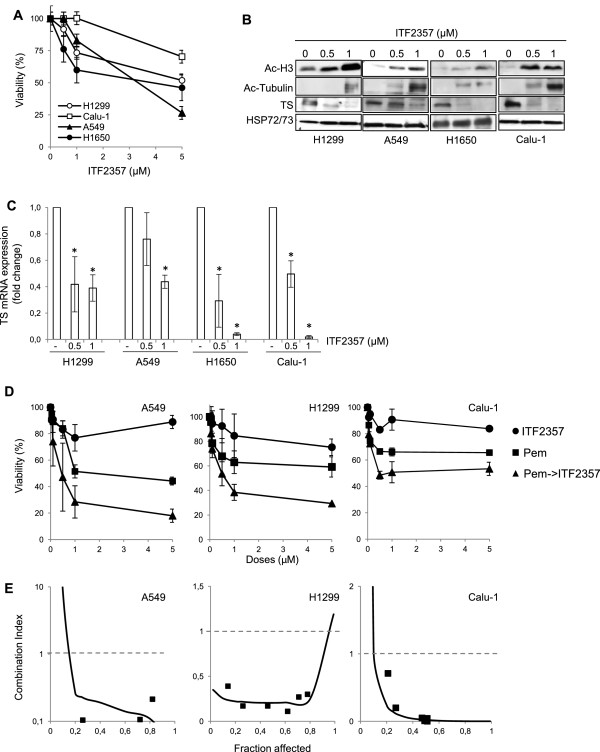
**Cytotoxic synergism of pemetrexed followed by ITF2357 in human NSCLC cell lines. (A)** Analysis of cell viability by MTT assay in the indicated NSCLC cell lines treated for 72 h with increasing concentrations of ITF2357. **(B)** Western blot analysis of acetylated histone H3 (Ac-H3), acetylated α-Tubulin (Ac-Tubulin) and TS protein expression in total cell lysates from the indicated NSCLC cell lines treated with increasing concentration of ITF2357 for 24 h. HSP72/73 expression was used as loading and transferring control. Western blots representative of two independent experiments with similar results are shown. **(C)** TS mRNA expression by quantitative RT-PCR in NSCLC cells treated with increasing concentration of ITF2357 for 24 h. Results are presented as the mean ± SD of two independent experiments. p values were calculated between control and treated cells (*p < 0.05). **(D)** Analysis of cell viability by MTT assay in the indicated NSCLC cell lines treated with ITF2357 and pemetrexed (Pem, drug ratio 1:1) alone or in combination. Treatment with pemetrexed for 24 h was followed by treatment with ITF2357 for 48 h. (●, ITF2357; ■, pemetrexed; ▲, combination). **(A,D)** The results are reported as "viability of drug-treated cells/viability of control cells" × 100 and represent the mean ± SD of three independent experiments performed in triplicate. **(E)** Interaction between pemetrexed followed by ITF2357 treatment evaluated on the basis of the Combination Index, which is plotted against fractional growth inhibition. Data are means of triplicates from experiments that were repeated three times.

We first investigated *in vitro* effects of the HDAC inhibitor ITF2357 in four NSCLC cell lines. ITF2357 dose-dependently inhibited the growth of all cell lines tested, with IC_50_ values ranging from 3 to 20 μM after 72 h of drug exposure (Figure 1A). Increased acetylation of both histone H3 and α-tubulin was observed by Western blot analysis after 24 h treatment with 0.5 and 1 μM ITF2357 in all cell lines tested (Figure 1B).

Next, we examined the levels of TS mRNA and protein expression following ITF2357 treatment. ITF2357 (0.5 and 1 μM) significantly reduced TS expression at both the mRNA (Figure 1C) and protein (Figure 1B) level in all four NSCLC cell lines.

Since TS expression may affect sensitivity to pemetrexed [13], we assessed whether ITF2357 could modulate pemetrexed cytotoxicity. To this purpose, combination studies were performed using a fixed-ratio experimental design (1:1) in H1299, A549 and Calu-1 cell lines using three different schedules: simultaneous treatment with the two drugs, ITF2357 followed by pemetrexed, and pemetrexed followed by ITF2357. As shown by growth inhibition curves (Additional file 1: Figure S1A) and analysis of drug interactions (Additional file 1: Figure S1B), simultaneous treatment with equipotent doses of pemetrexed and ITF2357 for 72h resulted in an antagonistic effect (H1299, CI = 1.7; A549, CI = 2.97, Calu-1: CI = 7.26) in all cell lines analyzed. The sequence of ITF2357 followed by pemetrexed had synergistic growth inhibitory effects in H1299 and Calu-1 cells (H1299: CI = 0.54; Calu-1: CI = 0.37), whereas it had an effect comparable to that of ITF2357 alone in A549 cells (Additional file 1: Figure S1A,B). Of note, the sequence of pemetrexed followed by ITF2357 was the most effective, with strikingly synergistic drug interactions and CI values < <1 in all cell lines studied: H1299, CI = 0.21; A549, CI = 0.15; Calu-1, CI = 0.02 (Figure 1D,E). A strong growth-inhibitory effect *in vitro* and a synergistic drug interaction was also observed in three other NSCLC cell lines (H1975, CI = 0.21; H460, CI = 0.12; H1650 CI = 0.59; Additional file 2: Figure S2A,B) treated with this schedule.

### Sequential treatment with ITF2357 enhances pemetrexed-induced apoptosis in NSCLC models

**Figure 2:**
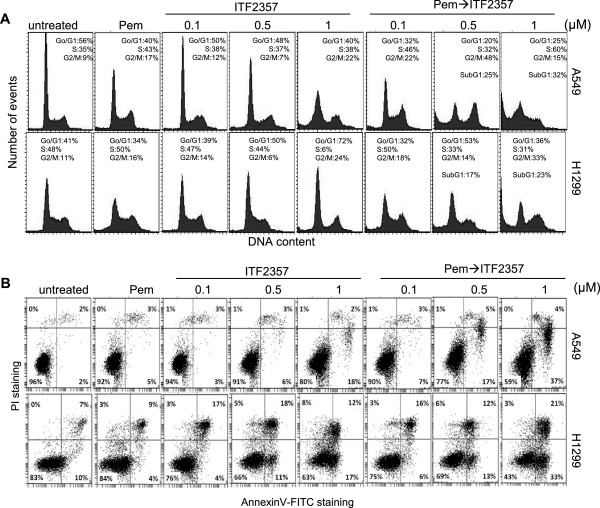
**Combination treatment with pemetrexed followed by ITF2357 leads to apoptosis induction in human NSCLC cell lines.** Flow cytometric analysis of **(A)** cell cycle distribution by PI staining, and **(B)** apoptotic cells by AnnexinV/PI staining in A549 and H1299 cell lines exposed to pemetrexed (Pem, 0.1 μM) or ITF2357 (from 0.1 to 1 μM) given alone or in combination (24 h pemetrexed followed by 48 h ITF2357). **(A)** The percentage of cells in sub-G1 and in the different cell cycle phases is shown. Where not reported, the percentage of cells in sub-G1 phase was less than 10%. Each panel is representative of three independent experiments with comparable results. **(B)** The percentage of AnnexinV+/PI- (early apoptotic cells, lower right), AnnexinV+/PI + (late apoptotic cells, upper right), AnnexinV-/PI- (viable cells, lower left) and AnnexinV-/PI + (necrotic cells, upper left), cells is shown.

Next, we explored the putative mechanisms of synergistic growth-inhibitory interactions induced by pemetrexed followed by ITF2357 in the A549 and H1299 representative cell lines. As shown in Figure 2A, less than 10% of cells with sub-G1 DNA content were detected after either drug alone; in contrast, when cells were exposed to pemetrexed followed by increasing concentrations of ITF2357, the percentage of sub-G1 cells dose-dependently increased up to 32% and 23% in A549 and H1299 cells, respectively. Apoptosis induction was confirmed by cytofluorimetric analysis of AnnexinV/PI staining (Figure 2B). Conversely, combined treatment with ITF2357 given simultaneously or before pemetrexed did not increase apoptosis induction in H1299 cells, as compared with treatment with individual drugs (data not shown). Consistently, pro-caspase 3 activation was observed in H1299 cells exposed to pemetrexed followed by ITF2357, but not in cells treated with either drug alone (Additional file 3: Figure S3F).

**Figure 3:**
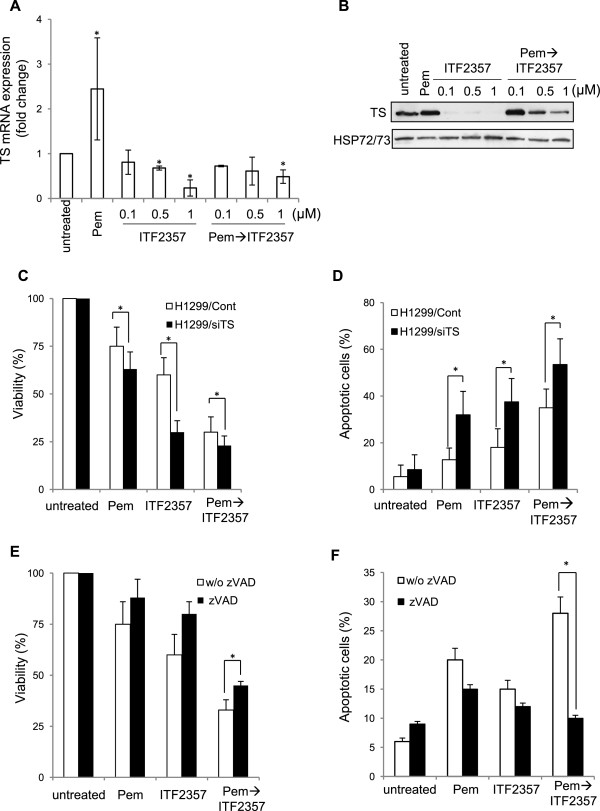
**Silencing of TS expression in H1299 cells enhances the apoptotic effect of pemetrexed and ITF2357 alone or in combination.** Analysis of TS mRNA **(A)** and protein **(B)** expression evaluated respectively, by quantitative RT-PCR **(A)** or Western blot analysis **(B)** in H1299 cells exposed to pemetrexed (Pem, 0.1 μM) or ITF2357 (from 0.1 to 1 μM) alone or in combination (24 h pemetrexed followed by 24 h ITF2357). **(A)** Results are presented as the mean ± SEM of 2 independent experiments. p values were calculated between untreated and treated cells (*p < 0.05). **(B)** HSP72/73 expression was used as loading and transferring control. Western blot representative of two independent experiments with similar results is shown. Analysis of **(C)** viable cells evaluated by CellTitleGLO, and **(D)** apoptotic cells evaluated by AnnexinV/PI staining in HI299 cells transiently transfected with control RNA interference (H1299/Cont) or RNA interference directed against TS (H1299/siTS) exposed to pemetrexed (0.1 μM) or ITF2357 (1 μM) alone or in combination (24 h pemetrexed followed by 48 h ITF2357). Analysis of **(E)** viable cells evaluated by CellTitleGLO and **(E)** apoptotic cells evaluated by AnnexinV/PI staining in HI299 exposed to ITF2357 or pemetrexed as reported in **C)** in absence or presence of the pan-caspase inhibitor zVAD (50 μM). **(C-F)** p values were calculated between untreated and treated cells (*p < 0.05). The results represent the mean ± SD of three independent experiments.

We also analyzed the effect of each agent, alone or in combination, on TS mRNA and protein expression in the H1299 cell line. After exposure to pemetrexed (24 h) followed by wash out of the drug (24 h), TS expression was upregulated at both mRNA (Figure 3A) and protein (Figure 3B) levels; conversely, ITF2357 profoundly downregulated TS mRNA and protein expression (Figure 1B,C and Figure 3A,B) and, in combination treatment, completely prevented pemetrexed-induced TS upregulation, when ITF2357 was used at high doses (Figure 3A,B). To determine if ITF2357-induced TS modulation is causally involved in the observed synergism, we next evaluated whether TS knockdown by a specific siRNA (Additional file 3: Figure S3A) affects ITF2357/pemetrexed-mediated apoptosis in H1299 cells. TS silencing sensitized cells to treatment with both agents, alone or in combination, in terms of both viability (Figure 3C) reduction and apoptosis induction (Figure 3D).

### Sequential treatment with pemetrexed followed by ITF2357 induces autophagy in NSCLC models

To assess the role of apoptosis in the growth inhibitory synergism observed with pemetrexed followed by ITF2357, we performed experiments in the absence or presence of the pan-caspase inhibitor zVAD. First, we analyzed apoptotic and non-apoptotic populations for active caspase-3, using A549 cells (Additional file 2: Figure S2C). We found that untreated, as well as pemetrexed-treated cells were primarily negative for the presence of active caspase-3, whereas about 20% of ITF2357 treated cells were positive for active caspase-3 staining. This percentage increased up to about 36% when cells were exposed to combined treatment and, as expected, the pan-caspase inhibitor zVAD completely inhibited caspase-3 activation. Similar results were observed in H1299 cells (data not shown). zVAD also almost completely inhibited apoptosis induced by combined treatment in H1299 cells, as demonstrated by the percentage of AnnexinV positive cells (Figure 3F), but had very limited, albeit statistically significant, effects on loss of viability induced by the combination (Figure 3E). Similar results were obtained with A549 cells (data not shown).

**Figure 4:**
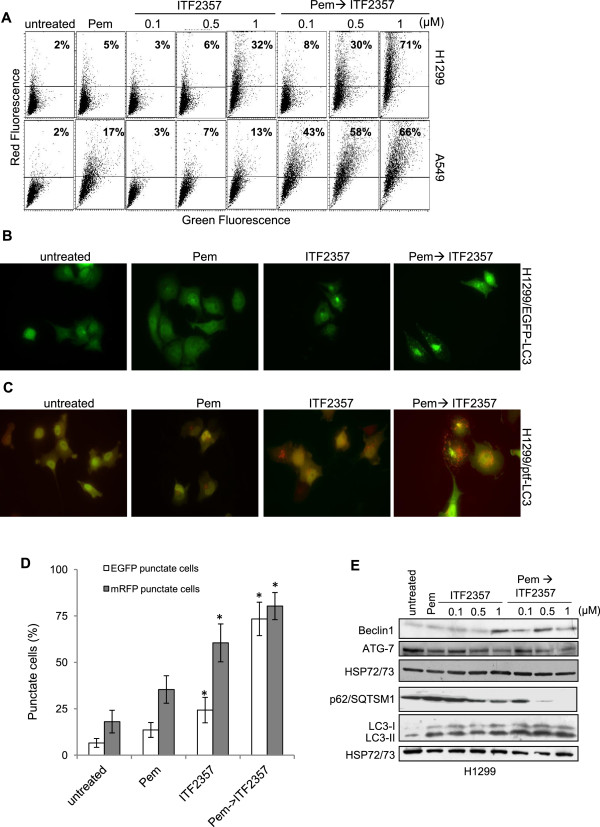
**Combination treatment with pemetrexed followed by ITF2357 leads to autophagy induction in human NSCLC cell lines. (A)** Flow cytometric analysis of acid vesicular organelles (AVOs) by acridine orange staining in H1299 and A549 cells exposed to pemetrexed (Pem, 0.1 μM) or ITF2357 (from 0.1 to 1 μM) alone or in combination (24 h pemetrexed followed by 48 h ITF2357). The percentage of cells with prominent red fluorescence, indicative of AVOs formation, is shown. Each panel is representative of three independent experiments with comparable results. Representative images of fluorescence microscopy in H1299 cells stably transfected with EGFP-LC3B vector (H1299/EGFP-LC3) **(B)**, and in H1299 cells stably transfected with ptf-LC3B vector (H1299/ptf-LC3) **(C)** exposed to pemetrexed (0.1 μM) or ITF2357 (1 μM) alone or in combination (24 h pemetrexed followed by 48 h ITF2357). **(D)** Quantification of cells positive for EGFP (green) and mRFP (red) autophagosomal structures treated as indicated in **B** and **C**. The results represent the mean ± SEM of three independent experiments. **(E)** Western blot analysis of p62/SQTSM1, Beclin1, ATG7, LC3B-I/II protein expression in H1299 cells treated as reported in **(A)**. HSP72/73 is shown as loading and transferring control.

Since both HDACi and pemetrexed can induce autophagic flux [28, 44, 45], we investigated autophagy as a potentially alternative mode of cell death activated by combined pemetrexed followed by ITF2357. As shown in Figure 4A, vital staining of H1299 and A549 cells with acridine orange showed prominent AVOs formation after exposure to pemetrexed followed by ITF2357, while a much lower percentage of AVOs positive cells was observed after treatment with individual drugs. Autophagy induction was further confirmed by analysis of microtubule associated protein I light chain 3 (LC3) redistribution in H1299 cells stably expressing an EGFP-LC3B fusion protein. As shown in Figure 4B,D, diffuse cytoplasmic distribution of green fluorescence was observed in both control and pemetrexed-treated cells, whereas an increase in the characteristic redistribution of EGFP-LC3B into punctate vesicular structures was observed in cells exposed to ITF2357 and, to a much greater extent, in cells exposed to sequential pemetrexed followed by ITF2357.

To obtain clearer results about the effect of drug combination on autophagic flux, we analysed the distribution and alteration of mRFP-GFP-LC3B fluorescent signals using H1299 cells stably expressing mRFP-EGFP-LC3 reporter (H1299/ptf-LC3) [37, 41] (Figure 4C,D). As GFP but not mRFP fluorescence is lost in acidic compartments, mRFP-GFP-LC3B labels nonacidic autophagosomes as yellow fluorescence (positive for both green and red) but acidic autophagolysosomes as red fluorescence only [41]. The increase of red fluorescence observed under ITF2357 and combination treatment indicates that autolysosome maturation proceeds normally. By contrast, as expected a prominent increase in the yellow fluorescence vesicles was observed in CQ-treated cells, indicating incomplete/impaired autophagosome maturation into lysosome (Additional file 4: Figure S4).

Consistent with these data, Western blot analysis performed in H1299 cells showed increased processing of the membrane-bound form of microtubule-associated protein LC3BII, decreased p62/SQTSM1 protein levels, and increased Beclin1 expression in ITF2357-treated cells, particularly after sequential exposure to pemetrexed followed by ITF2357 (Figure 4E). Conversely, no changes in ATG7 expression were observed after single and combined drug treatment (Figure 4E).

### Hierarchical activation of autophagy and apoptosis in response to sequential treatment with pemetrexed followed by ITF2357

**Figure 5:**
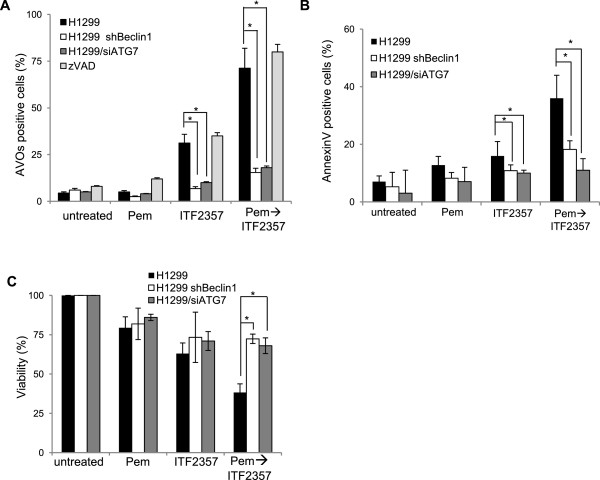
**Induction of autophagy is essential for cytotoxic effect of pemetrexed followed by ITF2357 in human NSCLC cell lines.** Analysis of **(A)** acid vesicular organelles (AVOs) by acridine orange, **(B)** apoptotic cells by AnnexinV/PI staining, and **(C)** cell viability by MTT assay in H1299 cells stably expressing control short hairpin RNA (H1299) or short hairpin RNA directed against Beclin1 (H1299 shBeclin1) or siRNA against ATG7 (H1299/siATG7) and exposed to pemetrexed (Pem, 0.1 μM) or ITF2357 (1 μM) alone or in combination treatment (24 h pemetrexed followed by 48 h ITF2357) in absence or presence of the pan-caspase inhibitor zVAD (50 μM).

As shown in Figures 2, 3, and 4, exposure of NSCLC cell lines to pemetrexed followed by ITF2357 induced both apoptosis and autophagy; moreover pharmacological caspase inhibition by zVAD completely abolished caspase 3 activation (Additional file 2: Figure S2) and apoptosis induction (Figure 3F), but only marginally affected cell viability (Figure 3E). Most importantly, zVAD had no effect on AVOs accumulation induced by drug combination (Figure 5A).

Since autophagy has been implicated in both inhibition and promotion of cell survival [46, 47], we investigated whether a functional relationship exists between apoptosis and autophagy, by silencing the expression of proteins acting at the early steps of autophagy, such as Beclin1 or ATG7, as a genetic approach to inhibit autophagy [41].

Selective knockdown of Beclin1 (Additional file 3: Figure S3B) substantially inhibited both AVOs formation (Figure 5A) and apoptosis induction, as measured by AnnexinV binding (Figure 5B), particularly in response to combined pemetrexed/ITF2357 treatment. Moreover, Western blot analysis performed in H1299/shBeclin1 cells exposed to different treatment with or without low concentrations of CQ, a late stage autophagic inhibitor, showed only slightly processing of LC3-II protein and p62 degradation in cells exposed to both single or combination treatment when compared to untreated cells, indicating that autophagic flux is regulated upstream by Beclin1. Of note, the addition of low doses of CQ dramatically promotes the LC3-II protein accumulation and recovery of p62 levels (Additional file 4: Figure S4B). Inhibition of both AVOs formation (Figure 5A) and apoptosis induction (Figure 5B), in response to combined pemetrexed/ITF2357 treatment was also observed following ATG7 silencing (Additional file 3: Figure S3C). Upon combination treatment selective knockdown of Beclin1 resulted in a decreased percentage of cells positive for active caspase-3 staining, when compared to control-transfected H1299 cells (Additional file 3: Figure S3F). Moreover, residual cells positive for active caspase-3 staining in H1299/shBeclin1 cells completely disappeared in the presence of pan-caspase inhibitor (data not shown). As a result, knockdown of either Beclin1 or ATG7 prevented the cytotoxic interaction between ITF2357 and pemetrexed, restoring the viability of cells exposed to the combination to levels comparable to those of cells exposed to ITF2357 or pemetrexed alone (Figure 5C). A similar rescue of cell viability after combined treatment was also observed when cells were exposed to 3MA, an early-stage autophagy inhibitor (Additional file 3: Figure S3D). As exposure to 3MA represents a condition that at high concentration can paradoxically induce autophagy by inhibiting class I PI3-kinase [48], the effect of 3MA on Akt/mTOR pathway has been investigated in H1299 cells. Western blot analysis demonstrated that phosphorylation of either Akt at Ser473 or mTOR was not affected by exposure to 3MA (Additional file 3: Figure S3E).

Taken together, these findings strongly indicate that the combination of pemetrexed followed by ITF2357 activates an autophagic flux, which, in turn, results in apoptosis induction and overall loss of cell viability.

### ITF2357 enhances pemetrexed cytotoxicity in patient-derived LCSC *in vitro*and NSCLC models *in vivo*

**Figure 6:**
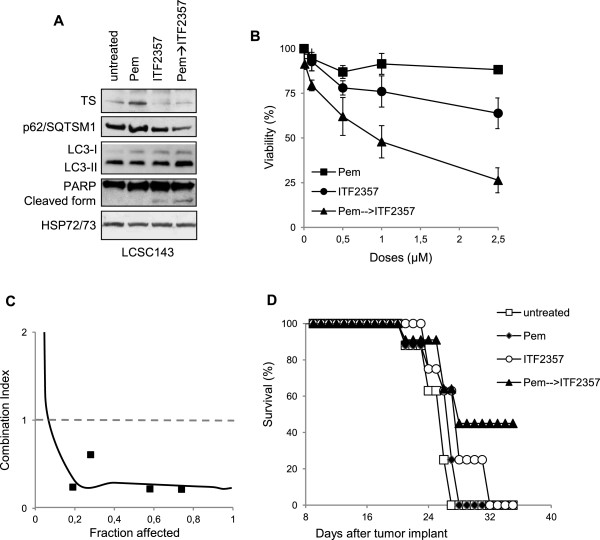
**Growth-inhibitory and molecular effects of sequential ITF2357/pemetrexed in LCSC** ***in vitro*** **and** ***in vivo*** **xenografts models. (A)** Western blot analysis of TS, p62/SQTSM1, LC3B-I/II and PARP proteins in total cell lysates from LCSC143 cell line exposed to pemetrexed (PEM, 0.1 μM) or ITF2357 (1 μM) alone or in combination (24 h pemetrexed followed by 48 h ITF2357). HSP72/73 expression was used as loading and transferring control. **(B)** Analysis of cell viability by CellTiter-Glo assay and **(C)** drugs interaction evaluated on the basis of the Combination Index, which is plotted against fractional growth inhibition in LCSC143 line treated with ITF2357 and pemetrexed (drug ratio 1:1) alone or in combination. Treatment with pemetrexed for 24 h was followed by treatment with ITF2357 for 48 h. (●, ITF2357; ■ , pemetrexed; ▲, combination) The results are reported as "viability of ITF2357-treated cells/viability of untreated cells" × 100 and represent the mean ± SD of three independent experiments. **(D)** *In vivo* response of H1650 xenograft to pemetrexed (1000 mg/kg/weekly) or ITF2357 (100 mg/kg day for 4 days) alone or in combination (pemetrexed, 1000 mg/kg/weekly followed by ITF2357, 100 mg/kg/day for 4 days).

The sensitivity to ITF2357 and pemetrexed alone or in combination and the modulation of markers of apoptosis and autophagy were also evaluated in patient-derived LCSC models *in vitro*. As reported in Additional file 5: Figure S5A, ITF2357 exposure dose-dependently reduced cell viability in four LCSC cell lines tested, although with different IC_50_ (0.1 and 0.5 μM in the highly sensitive LCSC136 and LCSC36 lines, as opposed to 5 and >5 μM in the relatively more resistant LCSC18 and LCSC143 cell lines, respectively). Increased histone H3 acetylation and PARP activation were observed in the LCSC136 cell line after exposure to ITF2357 (Additional file 3: Figure S3B). Consistent with the findings described above in NSCLC cell lines, ITF2357 decreased TS protein expression in the LCSC143 line (Figure 6A) and synergistically inhibited *in vitro* cell growth, when administered after pemetrexed exposure (Figure 6B,C). Combined treatment induced both apoptosis and autophagy, as evidenced by PARP cleavage, increased LC3B-II levels**,** and decreased p62/SQSTM1 protein expression (Figure 6A).

Finally, we investigated the *in vivo* efficacy of ITF2357 and pemetrexed alone or in combination. To this purpose, established H1650 xenografts in nude mice were treated with vehicle, pemetrexed (1000 mg/kg/weekly), ITF2357 (100 mg/kg/daily for 4 days), or sequential pemetrexed/ITF2357 (weekly pemetrexed followed by daily ITF2357 for 4 days, starting 24 h after pemetrexed administration). As reported in Figure 6D, pemetrexed and ITF2357 had minimal effect on mice survival. In contrast, sequential pemetrexed/ITF2357 administration inhibited tumor growth and prolonged mice survival, as compared to single agent administration. Similar to *in vitro* experiments, qRT-PCR analyses from tumor xenograft specimens evidenced a 2-fold increase and a 2-fold decrease in the median of TS mRNA relative to control after treatment with pemetrexed and ITF2357, respectively, while similar TS expression was observed in control and combined treatment groups. Such differences did not reach statistical significance (data not shown).

Similar results were also obtained in H1299 xenografts, with 35% inhibition of tumor growth with combined treatment, as compared to about 15% with single treatments, at day 35 after tumor cell injection (data not shown).

## Discussion

The present study sought to understand whether the modulation of TS expression induced by ITF2357-mediated HDAC inhibition might affect the response of NSCLC and LCSC-derived cell lines to pemetrexed. We found that the sequence of drug administration is critical in determining a synergistic interaction between pemetrexed and ITF2357 in both models. In particular, simultaneous treatment with the two drugs resulted in antagonistic effects, and ITF2357 followed by pemetrexed showed a synergistic effect only in some cell lines. On the contrary, a sequence-dependent, highly synergistic potentiation of pemetrexed activity was observed when ITF2357 was administered after pemetrexed exposure. Notably, such synergistic effect was evident in models of both adenocarcinoma and squamous cell carcinoma of the lung, a histologic subtype that is currently considered to be clinically resistant to pemetrexed [4], including patient-derived LCSC.

TS, one of the intracellular targets of pemetrexed, has been shown to be downmodulated by HDACi [23], through the Rb-E2F1-p21 axis, and hence may have a close association with pemetrexed efficacy. Compounds that modulate TS expression can potentially influence the activity of TS inhibitors and enhance the cytotoxicity of several drugs [49, 50]. We found that both TS mRNA and protein expression were downregulated by ITF2357 when administered alone or in combination with pemetrexed. Although these findings are consistent with our mechanistic hypothesis, the fact that TS downregulation by siRNA further enhanced apoptosis induction observed with single and combined pemetrexed treatment raises the possibility that other sites of pemetrexed action, such as aminoimidazolecarboxamide ribonucleotide formyltransferase, or glycinamide ribonucleotide transformylase, may also play a role in the observed cytotoxic interaction between pemetrexed and ITF2357.

Our study shows that the mechanism of cell death induced by the combined treatment involves both autophagy and apoptosis, and provides an interesting link between these two pathways and loss of cell viability. In particular, combined treatment induced a canonical molecular autophagic pathway dependent on Beclin1 and ATG7, involved in the nucleation and conjugation machinery, respectively. This process proceeds unimpeded, with the ultimate fusion between autophagosomes and lysosomes, resulting in degradation of the cargo of the autophagosomes by lysosomal hydrolases [30, 32]. Accumulating evidence reveals that autophagy and apoptosis can cooperate, antagonize or assist each other, thus differentially influencing cell fate [45–47, 51]. Using both pharmacologic and genetic approaches, we showed that these two mechanisms might act in concert to induce cell death upon sequential pemetrexed/ITF2357 treatment. In particular, when autophagy was repressed by Beclin1 or ATG7 knockdown, a decreased rate of apoptosis was detected in cells exposed to the drug combination. These results indicate that, in this specific context, autophagy positively controls apoptosis induction. Notably, genetic and pharmacological inhibition of late stages of autophagy, using a siRNA against LAMP2 or Chloroquine respectively, only marginally affected the effects of sequential ITF2357/pemetrexed on cell viability (data not shown). Conversely, the pan-caspase inhibitor zVAD did reduce apoptosis induction, but had little if any effect on autophagic markers and cell viability. In this context, it is important to note that the experimental data from viability measurements are not a function of sole caspases activation. On the other hand in many assays generally used for viability measurements the percentage cell viability could be significantly overestimated or underrated when the cells are committed to an autophagic pathway.

Overall, these results indicate that in NSCLC models sequential pemetrexed/ITF2357 causes a toxic form of autophagy, with consequent activation of the apoptotic program, which can lead to caspase-dependent apoptosis. From a mechanistic standpoint, we can speculate that the increased formation of acidic lysosomes, as the final end product of the autophagic flux, may lead to the release of active cathepsins or calpain proteases into the cytosol, where proapoptotic proteins such as BID can then be cleaved/activated [52]. It is also possible that Beclin1 and ATG5, which are required for the formation of autophagosomes, enhance susceptibility to apoptotic stimuli upon cleavage by proteases. Truncated proteins might translocate to mitochondria, and sensitize cells to apoptosis, possibly through the release of pro-apoptotic factors [53, 54]. A pro-apoptotic function for ATG12, the conjugation partner of ATG5, has been recently identified, being ATG12 required for caspase activation in response to a variety of apoptotic stresses [55]. The molecular mechanisms underlying the apoptotic function of the non-conjugated forms of ATG5 and ATG12 might be similar, as both proteins were shown to interact with anti-apoptotic members of the BCL2 family [54, 55]. Autophagy can also contribute to the induction of apoptosis activated by intrinsic stress signals through recruitment of caspase-8 to autophagosomes and consequent activation [56]. Finally, although protein degradation by autophagy is considered to be largely non-selective, an interesting concept for the direct regulation of apoptosis by autophagy is the selective targeting of apoptotic proteins for autophagic degradation. In this way, autophagy could shift the balance between anti- and pro-apoptotic factors, leading to initiation or inhibition of apoptosis [47, 55, 57].

*In vivo* experiments in nude mice provide a proof of the principle that a sequential schedule of pemetrexed followed by ITF2357 may indeed be developed for clinical testing, as the combination was able to substantially inhibit tumor growth and increase mice survival, with no increased toxicity. Such a sequential, intermittent, schedule of administration would have the advantage of maximizing the synergistic anti-tumor interactions between the two drugs, as indicated by *in vitro* modeling of different administration schedules, while simultaneously avoiding toxicities related to continuous HDACi administration [58].

## Conclusion

Our data indicate that combined treatment with pemetrexed followed by the HDACi ITF2357 has strikingly synergistic anti-tumor activity in NSCLC models. In particular, sequential administration of pemetrexed followed by ITF2357 was crucial in inducing growth-inhibitory synergism between the two drugs in all NSCLC models tested, at least in part due to ITF2357 ability to downregulate TS expression. Interestingly, while inhibition of initiation and autophagosome elongation blocked both AVOs formation and apoptosis induction, a pan-caspase inhibitor had no effect on AVOs accumulation induced by drug combination. These mechanistic studies highlighted a hierarchical activation of an autophagy program, which in turn results in the activation of caspase-dependent apoptosis, both of which contribute to loss of cell viability. Sequential pemetrexed followed by ITF2357 was also feasible and effective in xenograft models of NSCLC, supporting the possibility to develop such rational, mechanism-based combination schedule for the treatment of advanced NSCLC in the clinical setting.

## Electronic supplementary material

Additional file 1: Figure S1: **(A)** Analysis of cell viability by MTT assay in the indicated NSCLC cell lines treated with different schedules of ITF2357 and Pemetrexed (drug ratio 1:1). Cells were treated with each drug, either alone or in combination, as follows: 72 h ITF2357 and Pemetrexed, simultaneously (ITF2357 + PEM); 24 h ITF2357 followed by 48h Pemetrexed (ITF2357 -> PEM). The results are reported as "viability of drug-treated cells/viability of untreated cells" × 100 and represent the mean ± SD of three independent experiments. (■, Pemetrexed; ●, ITF2357; ▲, combination). **(B)** Interaction between Pemetrexed and ITF2357 treatment evaluated on the basis of the combination index (CI), which is plotted against fractional growth inhibition. Data are means of triplicates from experiments that were repeated three times. (PPTX 94 KB)

Additional file 2: Figure S2: **(A)** Analysis of cell viability by MTT assay in the indicated NSCLC cell lines treated with ITF2357 and Pemetrexed (drug ratio 1:1) alone or in combination (24 h Pemetrexed followed by 48 h ITF2357). (■, Pemetrexed; ●, ITF2357; ▲, combination). **(B)** Interaction between Pemetrexed and ITF2357 treatment evaluated on the basis of the combination index (CI), which is plotted against fractional growth inhibition. Cells were treated as reported in (A). Data are means of triplicates from experiments that were repeated three times. **(C)** Analysis of Active caspase-3 form by cytofluorimetric analysis in A549 cells exposed to pemetrexed (Pem, 0.1 μM) or ITF2357 (1 μM) alone or in combination treatment (24h pemetrexed followed by 48 h ITF2357) in absence or presence of the pan-caspase inhibitor zVAD (50 μM). (PPTX 156 KB)

Additional file 3: Figure S3: **(A)** TS mRNA expression by quantitative RT-PCR in H1299 cells transiently transfected with control RNA interference (H1299/Cont), or RNA interference directed against TS (H1299/siTS). Results are presented as the mean ± SD of 2 independent experiments. p values were calculated between control and treated cells (*p<0.05). Western blot analysis of Beclin1 **(B)** and ATG7 **(C)** protein expression in total cell lysates from H1299 cells stably expressing control short hairpin RNA (H1299 shCont) or short hairpin RNA directed against Beclin1 (H1299 shBeclin1) or ATG7 (H1299/siATG7). HSP72/73 expression was used as loading and transferring control. Western blots representative of two independent experiments with similar results are shown. **(D)** Analysis of viable cells evaluated by CellTiter-Glo, in HI299 exposed to Pemetrexed (PEM, 0.1 μM) or ITF2357 (1 μM) alone or in combined treatment (24 h Pemetrexed followed by 48 h ITF2357) in absence or presence of 3MA (1 mM). **(E)** Western blot analysis of phosphorylated forms of AKT and mTOR proteins in H1299 cells in absence or presence of 3MA (1 mM) for 48 h. HSP72/73 expression was used as loading and transferring control. Western blots representative of two independent experiments with similar results are shown. **(F)** Cytofluorimetric analysis of Active caspase-3 form in H1299 and H1299/shBeclin1 cells exposed to pemetrexed (Pem, 0.1 μM) or ITF2357 (1 μM) alone or in combination treatment (24 h pemetrexed followed by 48 h ITF2357). (PPTX 266 KB)

Additional file 4: Figure S4: **(A)** Representative images of autophagosomal structures by fluorescence microscopy in H1299 cells stably transfected with EGFP-LC3B vector (H1299/EGFP-LC3), and in H1299 cells stably transfected with ptf-LC3B vector (H1299/ptf-LC3) exposed to chloroquine (CQ, 25 mM) for 24 h. As GFP but not mRFP fluorescence is lost in acidic compartments, mRFP-GFP-LC3B labels non-acidic autophagosomes as yellow fluorescence (positive for both green and red) but acidic autophagolysosomes as red fluorescence only. **(B)** Western blot analysis of p62/SQTSM1 and LC3B-I/II protein expression in H1299/shBeclin1 cells treated with pemetrexed (Pem, 0.1 μM) or ITF2357 (0.5 μM) alone or in combination (24 h pemetrexed followed by 24 h ITF2357) in absence or presence of Chloroquine (CQ, 5 μM) for 18 h. β-actin is shown as loading and transferring control. Western blots representative of two independent experiments with similar results are shown. (PPTX 456 KB)

Additional file 5: Figure S5: **(A)** Analysis of cell viability in the indicated LCSC lines treated with ITF2357 for 72 h. The results are reported as "viability of treated cells/viability of untreated cells" × 100 and represent the mean ± SD of three independent experiments. **(B)** Western blot analysis of acetylated histone H3 (Ac-H3) and PARP protein expression in total cell lysates from LCSC136 cell line treated with increasing concentration of ITF2357 for 72 h. HSP72/73 expression was used as loading and transferring control. Western blots representative of two independent experiments with similar results are shown. (PPTX 129 KB)

## Abbreviations

NSCLC: Non-small cell lung cancer
HDACi: Histone deacetylase inhibitors
TS: Thymidylate Synthase
LCSC: Lung cancer stem-like cells
AVOs: Acidic vesicular organelles
CI: Combination index
MTT: 3-[4,5-dimethylthiazol-2-yl]-2,5-diphenyltetrazolium bromide inner salt.
