# Supplementary material for: Histone deacetylase inhibition synergistically enhances pemetrexed cytotoxicity through induction of apoptosis and autophagy in non-small cell lung cancer
Source: Mol Cancer. 2014 Oct 9;13:230. doi: 10.1186/1476-4598-13-230 (PMC4198757; doi:10.1186/1476-4598-13-230)
Supplement: Supplementary file 1 — Additional file 1: Figure S1: (A) Analysis of cell viability by MTT assay in the indicated NSCLC cell lines treated with different schedules of ITF2357 and Pemetrexed (drug ratio 1:1). Cells were treated with each drug, either alone or in combination, as follows: 72 h ITF2357 and Pemetrexed, simultaneously (ITF2357 + PEM); 24 h ITF2357 followed by 48h Pemetrexed (ITF2357 -> PEM). The results are reported as "viability of drug-treated cells/viability of untreated cells" × 100 and represent the mean ± SD of three independent experiments. (■, Pemetrexed; ●, ITF2357; ▲, combination). (B) Interaction between Pemetrexed and ITF2357 treatment evaluated on the basis of the combination index (CI), which is plotted against fractional growth inhibition. Data are means of triplicates from experiments that were repeated three times. (PPTX 94 KB) [file 12943_2014_1430_MOESM1_ESM.pptx]

## Slide 1
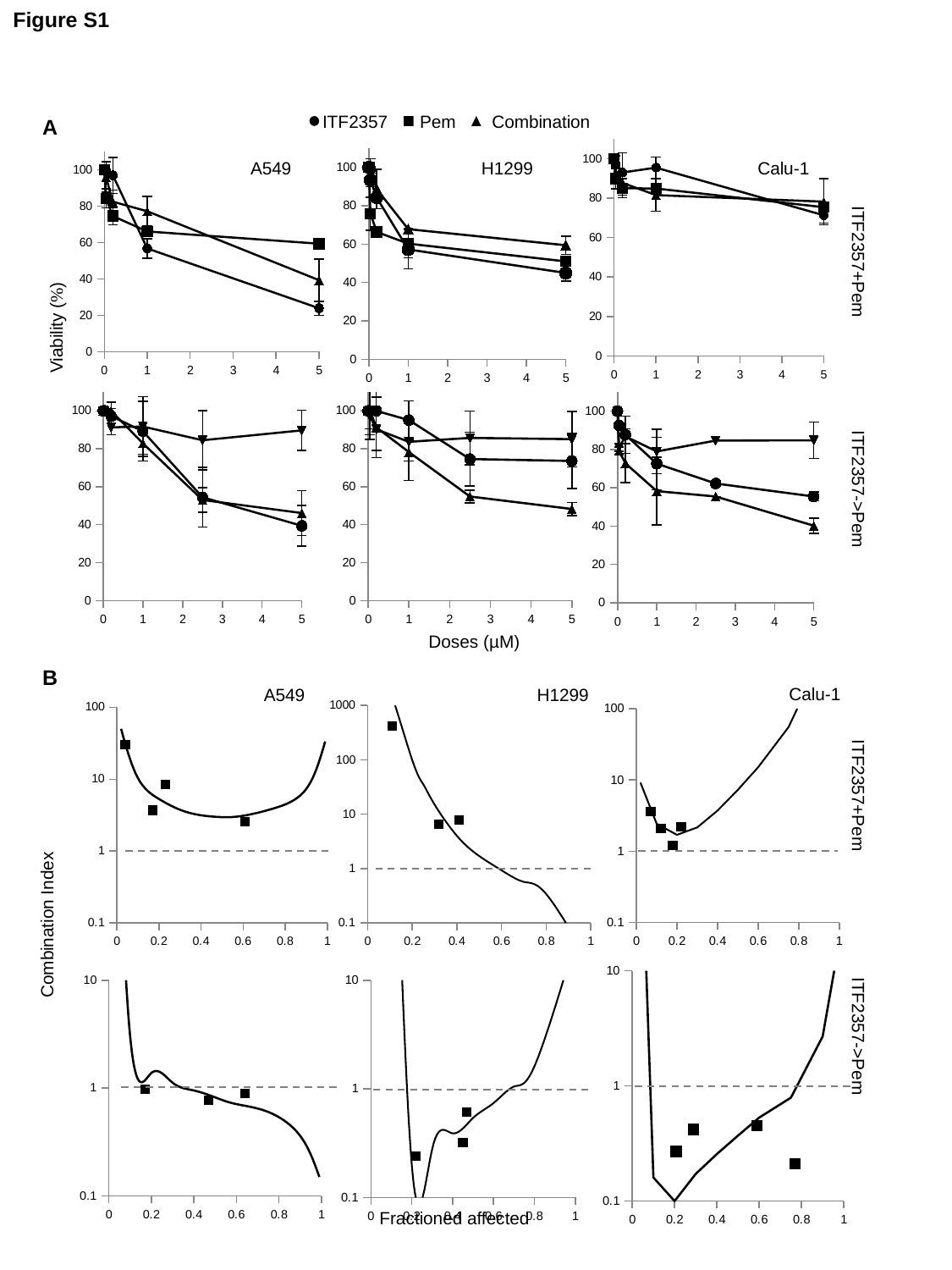

Figure S1
ITF2357
Pem
Combination
A
### Chart
| Category | | | |
|---|---|---|---|
### Chart
| Category | | | |
|---|---|---|---|
### Chart
| Category | | | |
|---|---|---|---|A549
H1299
Calu-1
ITF2357+Pem
Viability (%)
### Chart
| Category | ITF→CT | →PEM | ITF→PEM |
|---|---|---|---|
### Chart
| Category | ITF→CT | →PEM | ITF→PEM |
|---|---|---|---|
### Chart
| Category | ITF→CT | →PEM | ITF→PEM |
|---|---|---|---|ITF2357->Pem
Doses (µM)
B
Calu-1
A549
H1299
### Chart
| Category | | |
|---|---|---|
### Chart
| Category | | |
|---|---|---|
### Chart
| Category | | |
|---|---|---|ITF2357+Pem
Combination Index
### Chart
| Category | | |
|---|---|---|
### Chart
| Category | | |
|---|---|---|
### Chart
| Category | | |
|---|---|---|ITF2357->Pem
Fractioned affected
