# Supplementary material for: Histone deacetylase inhibition synergistically enhances pemetrexed cytotoxicity through induction of apoptosis and autophagy in non-small cell lung cancer
Source: Mol Cancer. 2014 Oct 9;13:230. doi: 10.1186/1476-4598-13-230 (PMC4198757; doi:10.1186/1476-4598-13-230)
Supplement: Supplementary file 2 — Additional file 2: Figure S2: (A) Analysis of cell viability by MTT assay in the indicated NSCLC cell lines treated with ITF2357 and Pemetrexed (drug ratio 1:1) alone or in combination (24 h Pemetrexed followed by 48 h ITF2357). (■, Pemetrexed; ●, ITF2357; ▲, combination). (B) Interaction between Pemetrexed and ITF2357 treatment evaluated on the basis of the combination index (CI), which is plotted against fractional growth inhibition. Cells were treated as reported in (A). Data are means of triplicates from experiments that were repeated three times. (C) Analysis of Active caspase-3 form by cytofluorimetric analysis in A549 cells exposed to pemetrexed (Pem, 0.1 μM) or ITF2357 (1 μM) alone or in combination treatment (24h pemetrexed followed by 48 h ITF2357) in absence or presence of the pan-caspase inhibitor zVAD (50 μM). (PPTX 156 KB) [file 12943_2014_1430_MOESM2_ESM.pptx]

## Slide 1
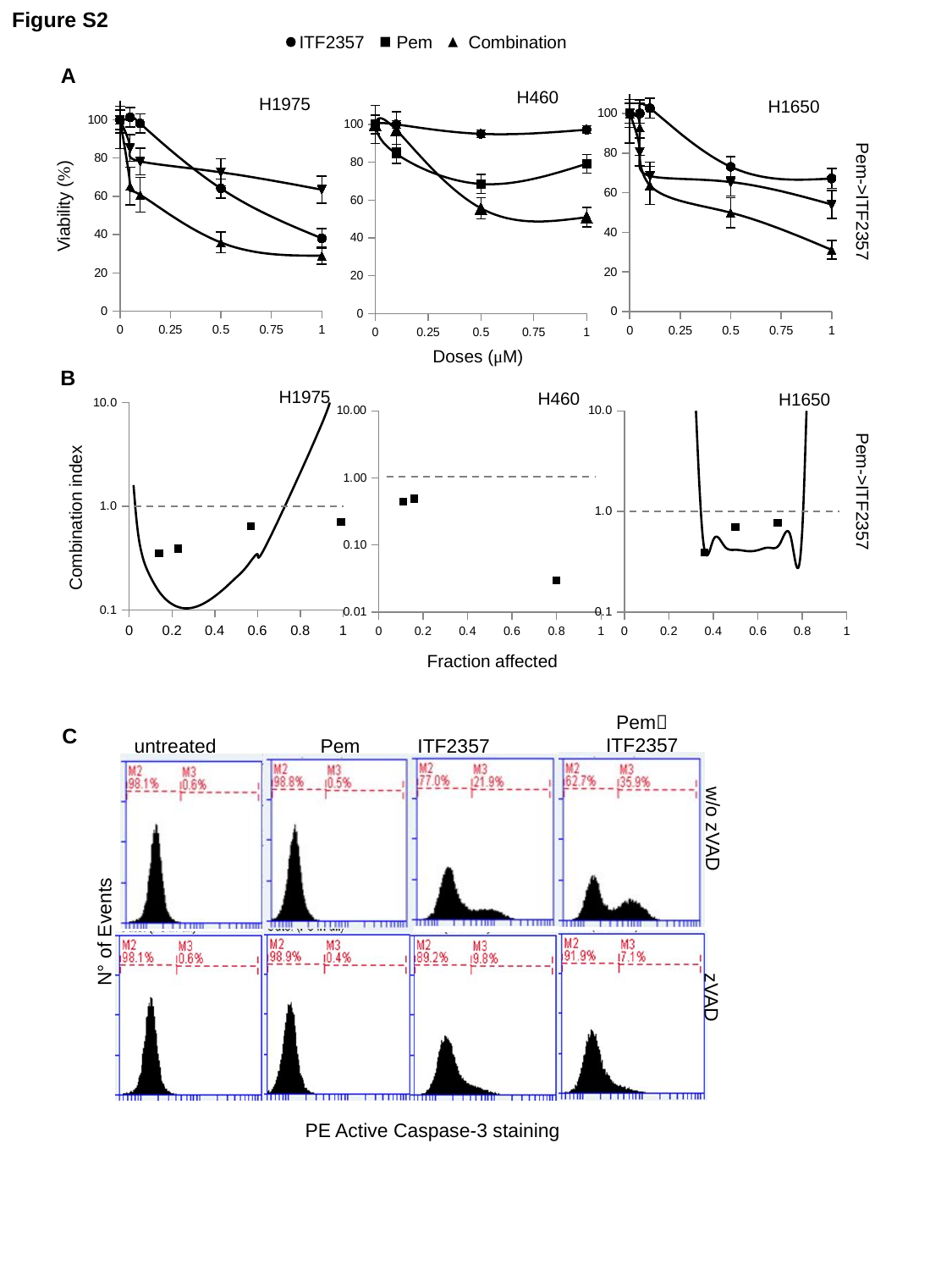

Figure S2
ITF2357
Pem
Combination
A
H460
### Chart
| Category | →ITF | PEM 24H | PEM 24H → ITF 72H |
|---|---|---|---|H1975
### Chart
| Category | →ITF | PEM 24H | PEM 24H → ITF 72H |
|---|---|---|---|H1650
### Chart
| Category | →ITF | PEM 24H | PEM 24H → ITF 72H |
|---|---|---|---|Viability (%)
Doses (μM)
Pem->ITF2357
B
H1975
H460
H1650
### Chart
| Category | h1975 | |
|---|---|---|
### Chart
| Category | | |
|---|---|---|
### Chart
| Category | | |
|---|---|---|Combination index
Fraction affected
Pem->ITF2357
Pem
ITF2357
ITF2357
untreated
Pem
w/o zVAD
N° of Events
zVAD
PE Active Caspase-3 staining
C
