# Supplementary material for: Histone deacetylase inhibition synergistically enhances pemetrexed cytotoxicity through induction of apoptosis and autophagy in non-small cell lung cancer
Source: Mol Cancer. 2014 Oct 9;13:230. doi: 10.1186/1476-4598-13-230 (PMC4198757; doi:10.1186/1476-4598-13-230)
Supplement: Supplementary file 3 — Additional file 3: Figure S3: (A) TS mRNA expression by quantitative RT-PCR in H1299 cells transiently transfected with control RNA interference (H1299/Cont), or RNA interference directed against TS (H1299/siTS). Results are presented as the mean ± SD of 2 independent experiments. p values were calculated between control and treated cells (*p<0.05). Western blot analysis of Beclin1 (B) and ATG7 (C) protein expression in total cell lysates from H1299 cells stably expressing control short hairpin RNA (H1299 shCont) or short hairpin RNA directed against Beclin1 (H1299 shBeclin1) or ATG7 (H1299/siATG7). HSP72/73 expression was used as loading and transferring control. Western blots representative of two independent experiments with similar results are shown. (D) Analysis of viable cells evaluated by CellTiter-Glo, in HI299 exposed to Pemetrexed (PEM, 0.1 μM) or ITF2357 (1 μM) alone or in combined treatment (24 h Pemetrexed followed by 48 h ITF2357) in absence or presence of 3MA (1 mM). (E) Western blot analysis of phosphorylated forms of AKT and mTOR proteins in H1299 cells in absence or presence of 3MA (1 mM) for 48 h. HSP72/73 expression was used as loading and transferring control. Western blots representative of two independent experiments with similar results are shown. (F) Cytofluorimetric analysis of Active caspase-3 form in H1299 and H1299/shBeclin1 cells exposed to pemetrexed (Pem, 0.1 μM) or ITF2357 (1 μM) alone or in combination treatment (24 h pemetrexed followed by 48 h ITF2357). (PPTX 266 KB) [file 12943_2014_1430_MOESM3_ESM.pptx]

## Slide 1
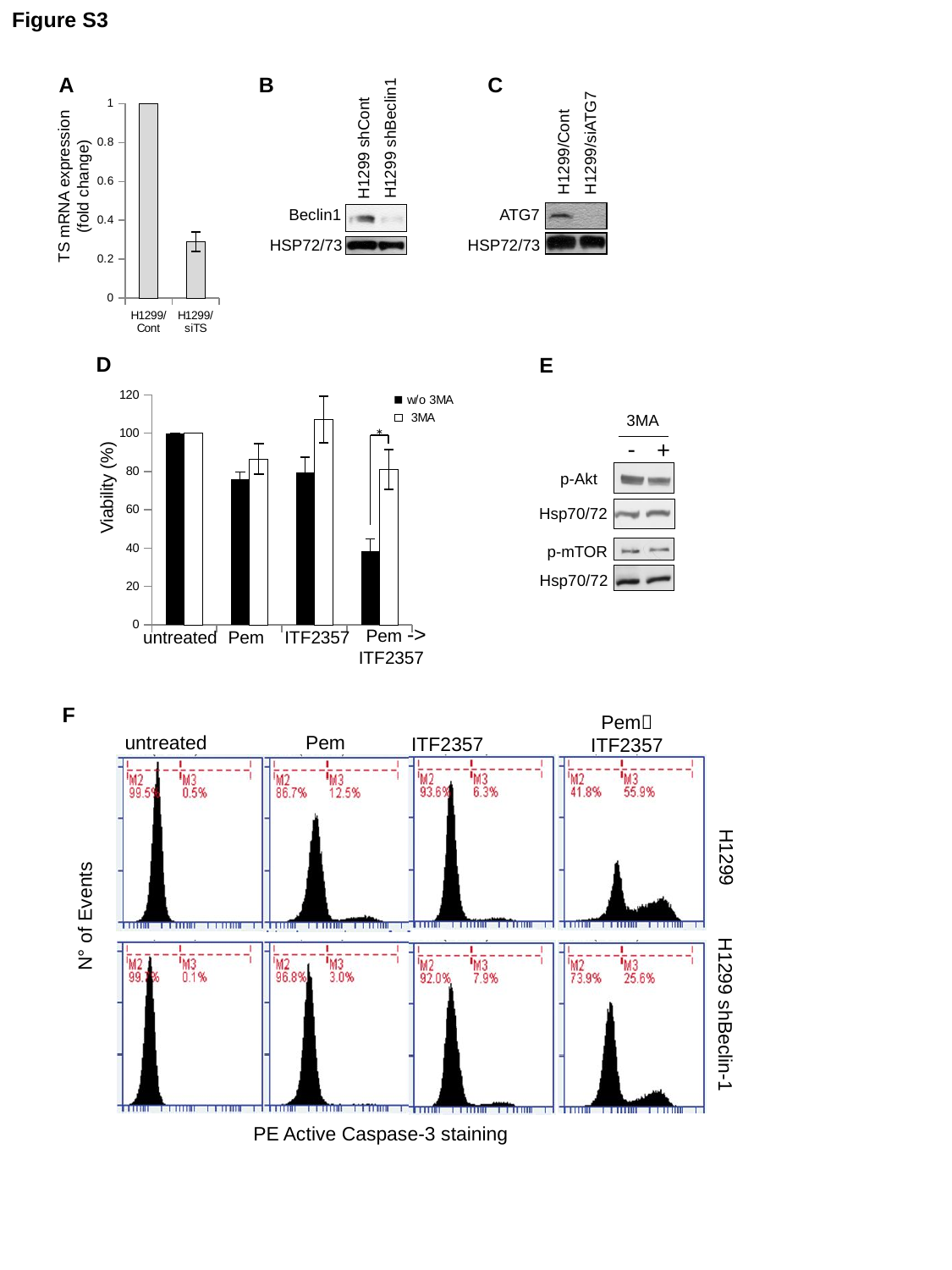

Figure S3
A
B
C
### Chart
| Category | |
|---|---|
| H1299/Cont | 1.0 |
| H1299/siTS | 0.2898427377021559 |H1299 shBeclin1
H1299/siATG7
H1299 shCont
H1299/Cont
TS mRNA expression
 (fold change)
Beclin1
ATG7
HSP72/73
HSP72/73
D
### Chart
| Category | w/o 3MA | 3MA |
|---|---|---|
| untreated | 100.0 | 100.0 |
| Pem | 76.13171640476031 | 86.60522144909017 |
| ITF2357 | 79.36818527508352 | 107.13568145109348 |
| Pem->ITF2357 | 38.33333333333334 | 81.00758391077143 |*
 Viability (%)
Pem -> ITF2357
Pem
ITF2357
untreated
E
3MA
- +
p-Akt
Hsp70/72
p-mTOR
Hsp70/72
F
Pem
ITF2357
ITF2357
untreated
Pem
H1299
N° of Events
H1299 shBeclin-1
PE Active Caspase-3 staining
