# Supplementary material for: Histone deacetylase inhibition synergistically enhances pemetrexed cytotoxicity through induction of apoptosis and autophagy in non-small cell lung cancer
Source: Mol Cancer. 2014 Oct 9;13:230. doi: 10.1186/1476-4598-13-230 (PMC4198757; doi:10.1186/1476-4598-13-230)
Supplement: Supplementary file 4 — Additional file 4: Figure S4: (A) Representative images of autophagosomal structures by fluorescence microscopy in H1299 cells stably transfected with EGFP-LC3B vector (H1299/EGFP-LC3), and in H1299 cells stably transfected with ptf-LC3B vector (H1299/ptf-LC3) exposed to chloroquine (CQ, 25 mM) for 24 h. As GFP but not mRFP fluorescence is lost in acidic compartments, mRFP-GFP-LC3B labels non-acidic autophagosomes as yellow fluorescence (positive for both green and red) but acidic autophagolysosomes as red fluorescence only. (B) Western blot analysis of p62/SQTSM1 and LC3B-I/II protein expression in H1299/shBeclin1 cells treated with pemetrexed (Pem, 0.1 μM) or ITF2357 (0.5 μM) alone or in combination (24 h pemetrexed followed by 24 h ITF2357) in absence or presence of Chloroquine (CQ, 5 μM) for 18 h. β-actin is shown as loading and transferring control. Western blots representative of two independent experiments with similar results are shown. (PPTX 456 KB) [file 12943_2014_1430_MOESM4_ESM.pptx]

## Slide 1
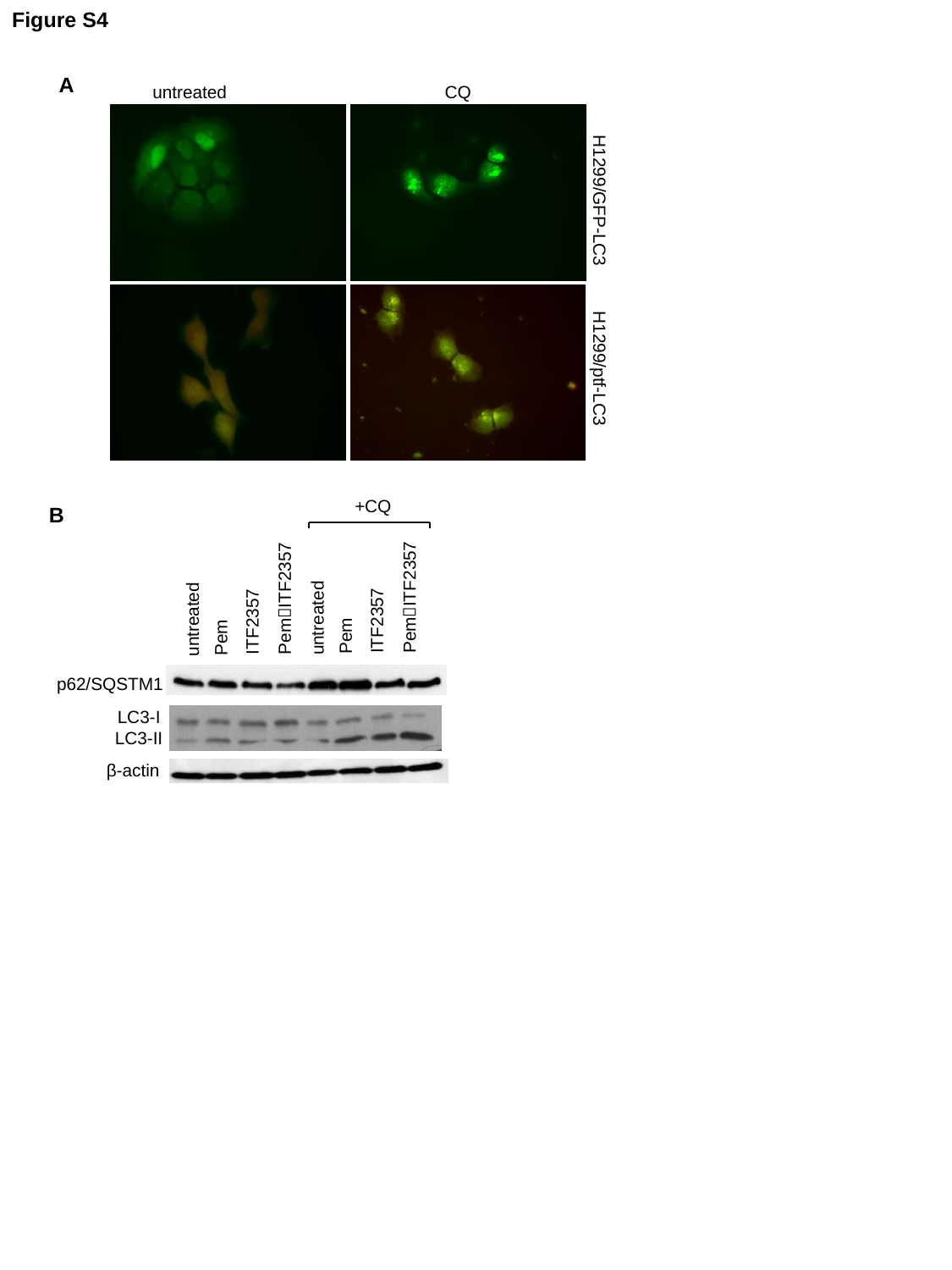

Figure S4
A
CQ
untreated
H1299/GFP-LC3
H1299/ptf-LC3
+CQ
PemITF2357
PemITF2357
ITF2357
ITF2357
untreated
untreated
Pem
Pem
p62/SQSTM1
LC3-I
LC3-II
β-actin
B
