# Supplementary material for: Histone deacetylase inhibition synergistically enhances pemetrexed cytotoxicity through induction of apoptosis and autophagy in non-small cell lung cancer
Source: Mol Cancer. 2014 Oct 9;13:230. doi: 10.1186/1476-4598-13-230 (PMC4198757; doi:10.1186/1476-4598-13-230)
Supplement: Supplementary file 5 — Additional file 5: Figure S5: (A) Analysis of cell viability in the indicated LCSC lines treated with ITF2357 for 72 h. The results are reported as "viability of treated cells/viability of untreated cells" × 100 and represent the mean ± SD of three independent experiments. (B) Western blot analysis of acetylated histone H3 (Ac-H3) and PARP protein expression in total cell lysates from LCSC136 cell line treated with increasing concentration of ITF2357 for 72 h. HSP72/73 expression was used as loading and transferring control. Western blots representative of two independent experiments with similar results are shown. (PPTX 129 KB) [file 12943_2014_1430_MOESM5_ESM.pptx]

## Slide 1
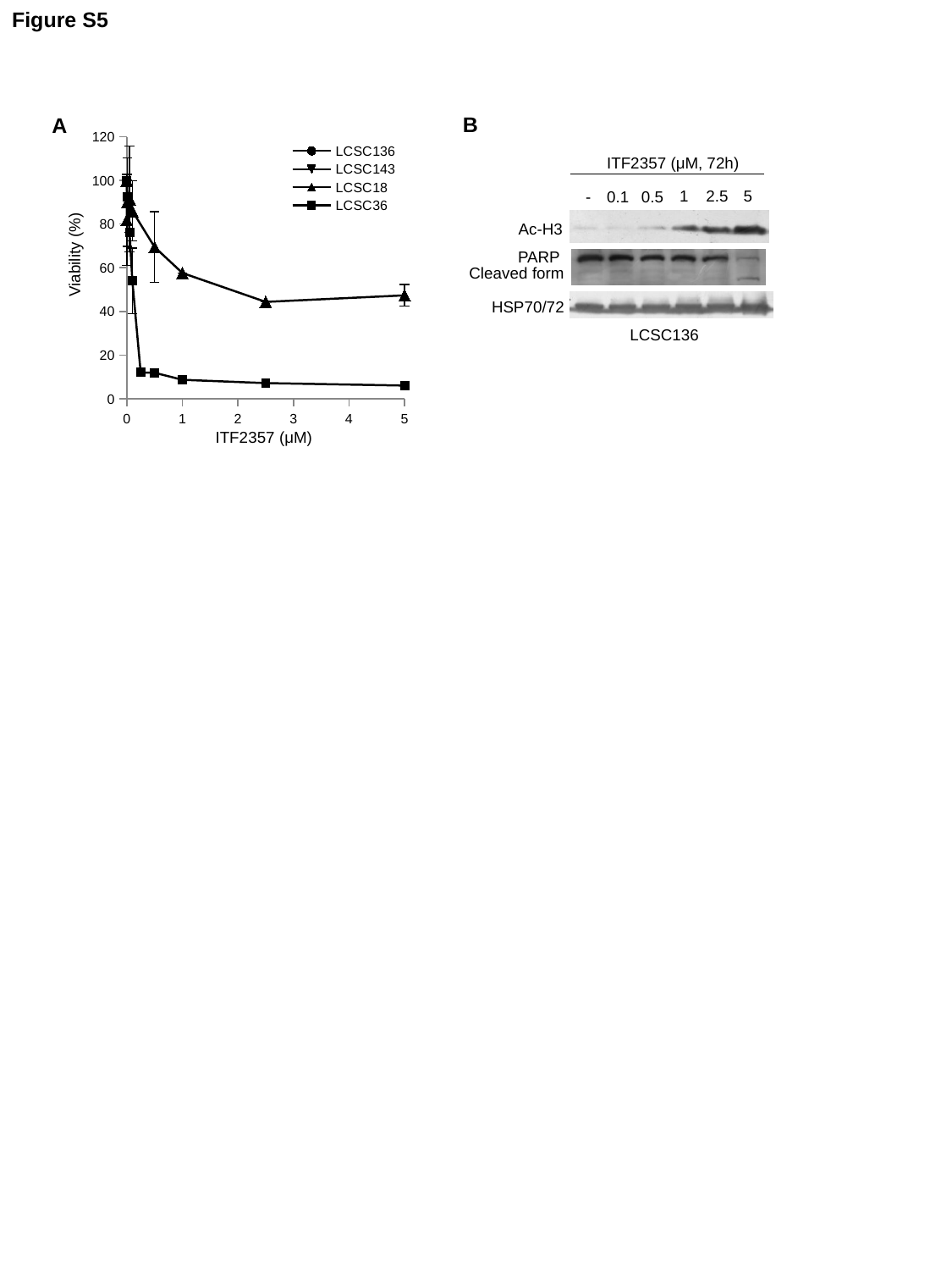

Figure S5
B
A
### Chart
| Category | LCSC136 | LCSC143 | LCSC18 | LCSC36 |
|---|---|---|---|---|ITF2357 (μM, 72h)
1
2.5
5
-
0.1
0.5
Ac-H3
Viability (%)
PARP
Cleaved form
HSP70/72
LCSC136
ITF2357 (μM)
